# Anxiety in heart failure patients and its association with NYHA class

**DOI:** 10.1192/j.eurpsy.2021.503

**Published:** 2021-08-13

**Authors:** F. Costa, S. Martins, E. Moreira, J. Silva Cardoso, L. Fernandes

**Affiliations:** 1 Department of Clinical Neuroscience And Mental Health and Center for health technology and services research (cintesis), Faculty of Medicine - University Porto, Porto, Portugal; 2 Center For Health Technology And Services Research (cintesis), Faculty of Medicine - University Porto, Porto, Portugal; 3 Department of Medicine, Faculty of Medicine - University Porto, Porto, Portugal; 4 Department of Cardiology, Centro Hospitalar Universitário S. João (CHUSJ), Porto, Portugal; 5 Psychiatry Service, Centro Hospitalar Universitário de São João, Porto, Portugal, Porto, Portugal

**Keywords:** Anxiety, heart failure, NYHA class

## Abstract

**Introduction:**

Heart failure (HF) is a worldwide public health problem and the main cause of morbidity and mortality in older people. Previous studies have demonstrated that psychological symptoms are associated with worse cardiovascular outcomes. Nevertheless, the research regarding the association between anxiety and HF is still scarce.

**Objectives:**

To analyse the levels of anxiety in HF patients and its association with New York Heart Association (NYHA) class in HF patients.

**Methods:**

This study takes part of a wider project named Deus Ex-Machina project (NORTE-01-0145-FEDER-00026). HF patients were recruited from an outpatient clinic at a University Hospital. Patient with inability to communicate, with severe visual impairment or with NYHA class IV were excluded. Sociodemographic data and NYHA class were recorded. Anxiety was assessed using the Generalized Anxiety Disorder-7 (GAD-7).

**Results:**

Overall, 136 patients were included, with a mean age of 57(±13) years old. Most of them were men (66%) and married (76%), with mean education of 8 years (±4). Regarding NYHA class, 36%, 49% and 15% were at class I, II and III, respectively. The mean GAD-7 total score was 6.4 (±5.2) and 32% of patients showed moderate to severe anxiety symptoms. No association between the NYHA functional class and anxiety was found (p=0.106).

**Conclusions:**

The results reveal that anxiety is frequent among HF patients. However, as found in previous studies, it was not associated with more severe HF symptoms. The coexistence of HF and anxiety deserves further studies, in order to build a better understanding of this association.

